# Tumor vascular normalization as a strategy to potentiate effectiveness of therapeutic vaccines

**DOI:** 10.1186/2051-1426-2-S3-P204

**Published:** 2014-11-06

**Authors:** Benedetto Farsaci, Renee N Donahue, Michael A Coplin, Italia Grenga, Lauren Lepone, Alfredo A Molinolo, James W Hodge

**Affiliations:** 1 Laboratory of Tumor Immunology and Biology, CCR, NCI, NIH, Bethesda, MD, USA; 2 NCI/CCR/LTIB, Bethesda, USA; 3 Laboratory of Tumor Immunology and Biology, CCR, NCI, NIH, Atlanta, MD, USA; 4 Oral and Pharyngeal Cancer Branch, NIDCR, NIH, Bethesda, MD, USA

## Purpose

The effectiveness of cancer immunotherapy can be compromised when immune cells cannot penetrate the tumor. We hypothesize that combining an antiangiogenic tyrosine kinase inhibitor (TKI) with a cancer vaccine can increase antitumor response by targeting 3 elements of the tumor microenvironment (TME): (a) tumor endothelial cells, which can lead to vascular normalization; (b) tumor cells, which can reduce tumor compactness and allow collapsed vessels to reopen; (c) tumor infiltrating immune cells, which can increase the frequency and function of effector immune elements, i.e. tumor-infiltrating lymphocytes (TILs) and anti-tumor myeloid cells, and decrease number and function of immune suppressor cells, i.e. T-regulatory lymphocytes (Tregs), myeloid-derived suppressor cells (MDSCs), and immune suppressive tumor-associated macrophages (TAMs).

## Methods

Animal studies were conducted using antiangiogenic TKIs (sunitinib or sorafenib) in combination with recombinant vaccines in 2 murine tumor models: colon carcinoma (MC38-CEA) and breast cancer (4T1). Tumor vasculature was measured by immunohistochemistry for the identification of mature (CD31+), immature/proliferating (CD105+), and monocytic (CD11b+) endothelial cells. Tumor oxygenation, tight junctions, compactness, and tissue pressure were assessed after treatment with either TKI alone, vaccine alone, or combination. In addition, the frequency and phenotype of TILs, MDSCs, and TAMs were evaluated.

## Results

The combination of either TKI with vaccine decreased tumor vasculature, compactness, tight junctions, and pressure, leading to vascular normalization and increased tumor oxygenation. Combination therapy increased TILs, including tumor antigen-specific CD8 T-cells, and elevated expression of the activation markers FAS-L, CXCL-9, CD31, and CD105 in MDSCs and TAMs, leading to reduced tumor volumes and an increased number of tumor-free animals.

## Conclusions

The improved antitumor activity induced by combining antiangiogenic TKIs with vaccine may be due to the immunologic effects of vascular normalization within tumors. The improved vascular perfusion facilitated the penetration, while the increased tumor oxygenation increased the activation of lymphoid and myeloid cells in the TME. These microarchitectural and immunologic changes in the TME acted in synergy with the tumor-associated antigen-specific immune activation elicited by therapeutic vaccination. Therapies that alter tumor architecture can thus have a dramatic impact on the effectiveness of cancer immunotherapy.

